# Metals Recovery from Artificial Ore in Case of Printed Circuit Boards, Using Plasmatron Plasma Reactor

**DOI:** 10.3390/ma9080683

**Published:** 2016-08-10

**Authors:** Jakub Szałatkiewicz

**Affiliations:** Measuring Systems Division, Industrial Research Institute for Automation and Measurements, Warszawa PL02-486, Poland; jakub.szalatkiewicz@gmail.com; Tel.: +48-22-874-0278

**Keywords:** metals recovery, artificial ore, waste treatment, printed circuit boards, plasma technology, plasmatron, recycling

## Abstract

This paper presents the investigation of metals production form artificial ore, which consists of printed circuit board (PCB) waste, processed in plasmatron plasma reactor. A test setup was designed and built that enabled research of plasma processing of PCB waste of more than 700 kg/day scale. The designed plasma process is presented and discussed. The process in tests consumed 2 kWh/kg of processed waste. Investigation of the process products is presented with their elemental analyses of metals and slag. The average recovery of metals in presented experiments is 76%. Metals recovered include: Ag, Au, Pd, Cu, Sn, Pb, and others. The chosen process parameters are presented: energy consumption, throughput, process temperatures, and air consumption. Presented technology allows processing of variable and hard-to-process printed circuit board waste that can reach up to 100% of the input mass.

## 1. Introduction

Increasing digitization of appliances and machines equips them with electronic and electrical printed circuit boards (PCB). A worn out appliance becomes hazardous waste that needs to be handled adequately. Collected waste of electrical and electronic equipment (WEEE) is processed in special plants where printed circuit boards are removed and sent for further specialized processing. On the other hand, mass production of electric and electronic equipment requires large amounts of non-renewable resources, including precious metals. That is why it is important to develop new effective ways of recycling electronic printed circuit board waste, as they become a “renewable” resource that can supply recycled metals for new production. 

Waste of electrical and electronic equipment is a global concern. In the 27 EU countries, it is estimated that the mass of produced WEEE in 2005 was 8.3–9.1 million Mg (tonne), 25% of which is collected and processed, while the remaining 75% is not registered and does not occur at collection points [1]. Such state of the waste management system can be caused by the lack of processing capacities and suitable technologies which can utilize WEEE effectively. The amount of WEEE increases continuously [2]. Moreover, the European Commission proposes increasing the collection targets from 4 kg/capita to 65% of the average mass of electrical and electronic equipment placed on the market [3]. WEEE has to be disposed of, but it also can become a source of valuable resources. 

Among different methods available for processing of PCB waste, this paper presents novel and unique high-temperature pyrometallurgical process based on a non-transferred plasma application. The presented approach allows high efficiency of metals recovery from PCB waste including precious metals in the form of copper-rich ingots in the one-stage process. Also, no toxic waste is generated by the process and the PCB waste input can be up to 100% PCB.

### 1.1. Technologies for Processing Printed Circuit Board Waste

There are two main approaches to PCB treatment and metals recovery: pyrometallurgical smelting of waste and mechanical processing (milling). However, there are also other approaches allowing processing of PCB, for example incineration or pyrolysis [4]. Pyrolysis is applied in one industrial scale plant only, but many works, including those of authors, indicate that it is an interesting approach that allows concentration of metals and preparation of PCB feedstock for further recovery/processing. Unfortunately, the main drawback of the pyrolysis method is the use of pyrolysis “liquids”, and gaseous products that are generated in the process. Those liquids and gases contain significant amounts of brominated hydrocarbons and are extremely toxic, as bromine concentration in a PCB can reach 5%–6% [5], which it is released in the pyrolysis process [6]. Despite those drawbacks, the solid residue form pyrolysis of PCB is a good concentrate of metals mixed with carbon, without the violate part. Apart from the pyrolysis, hydrometallurgical technologies are applied and investigated, however not on the industrial scale as pyrometallurgy is. The hydrometallurgical processes are interesting as they allow recovery, segregation, and refining of metals from PCB that were released from the PCB epoxy resin enclosure during the pyrolysis process. There are many hydrometallurgical approaches to metals recovery, some based on cyanide solutions [7], other on acids application [8], and many other methods [9]. The main advantage of the hydrometallurgical processes is their capability for selectivity in the recovery of metals. On the other hand, hydrometallurgical processes generate liquid waste that needs to be utilized [10] or neutralized before being be disposed of. Very interesting works are carried out to detect and separate precious metals from PCB waste [11]. Those new methods are being developed to allow recovery—even of trace amounts—of precious metals. Such an approach increases the metals recovery efficiency and increases the economy of the recycling process. 

It is essential to choose the right approach for recovery of metals from given feedstock, for example PCB, that, on one hand, will guarantee high recovery of metals, and on the other, will not produce additional problematic waste. That is why the pyrometallurgical processes are favorable in metals recovery from PCB, and other processes are applied in only specific cases or as a part of the entire recovery process. 

**Figure 1:**
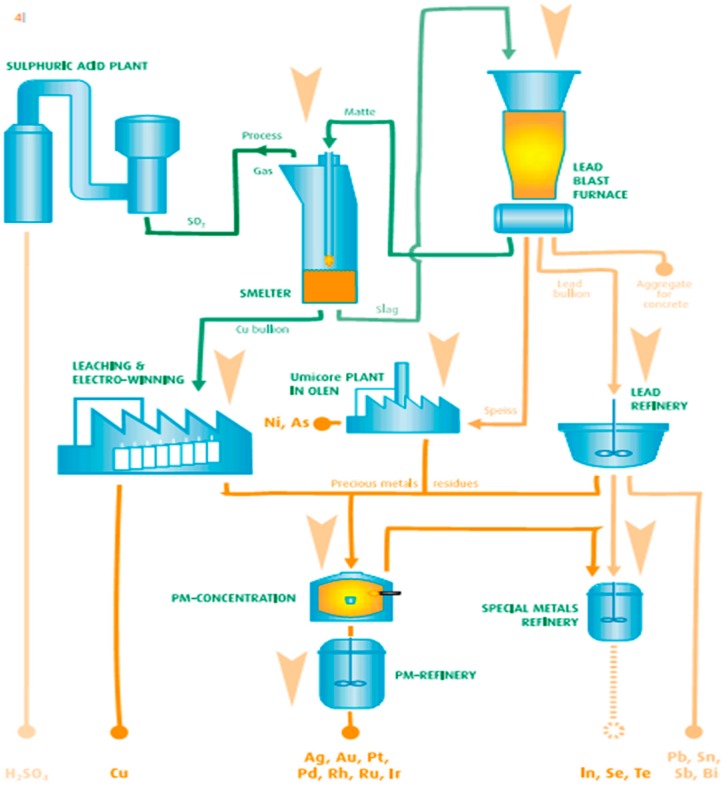
Umicore waste-to-metals technology [12].

Currently in Europe only a few plants process electronic printed circuit boards using pyrometallurgical processes. Those plants process PCB from the entirety of Europe. However, the waste of electric and electronic printed circuit boards is only a part of the total input in those installations, as they prefer homogenous industrial waste over PCB waste from WEEE. Integrated pyrometallurgical smelters can process up to 300,000 Mg/year [12] of waste, and recover up to 95% of precious metals and Cu. Pyrometallurgical technologies process waste by incinerating organic substances, and smelting the metals and slag from it. Smelted metal concentrates are further processed in a specialized unit and then refined to recover pure metals. A diagram of the Umicore process is presented in the Figure 1. 

Another way of processing waste of printed circuit boards is through the use of mechanical methods based on fine milling, and multi-step segregation of such powdered materials. Mechanical processing allows recovery of aluminium, copper (heavy category), plastics, ferrous metals, glass, and dusts. A disadvantage of those methods is the loss of precious metals [13] and contamination of recovered materials; the recovered and segregated resources need to be further processed using pyrometallurgical or chemical processes to recover pure metals and remove the contaminants. Mechanical methods are widely used to process WEEE and recover Al, Fe, Cu (brass), plastics, and printed circuit boards. Then, those resources are sold to companies that process and purify them to the form of pure metal products. As for the category of PCB segregated from WEEE, this resource is sold to smelting plants for recovery of metals. As presented in this paper, plasma technology can become an alternative for the centralized smelting technologies described above. 

### 1.2. Artificial Ore Characterization—Waste Printed Circuit Boards

Waste printed circuit boards are found in almost all electric and electronic equipment waste. After their removal, they are often segregated by precious metals content. This classification narrows down the variety of PCB only to Au-rich and Au-poor ones. Further classifications are uncommon, and despite segregation being carried out according to this classification, one lot can vary from another in a broad range. For example, PCB varies by the year of production and its type; there are also different PCB types in tools, radios, TVs, PCs, military equipment, etc. To analyze such a variety of PCB it is necessary to focus on their similarity. Resemblance can be found in PCB elements that constitute their mass. 

Compositions of printed circuit boards are presented in Table 1 and Table 2. Data from Table 1 and Table 2 indicate that about 30% of PCB mass is made up of metal elements. The rest of the PCB is inorganic filler (about 37%) and organic substances (31%). Using this information as a point of reference for the smelting process, it is estimated that 1/3 of the PCB mass is potentially available for recovery of metals, 1/3 will become slag, and 1/3 will be incinerated. Due to the variety of PCB, this rough estimation is precise enough for the needs of this analysis [5].

## 2. Materials and Methods 

**Figure 2:**
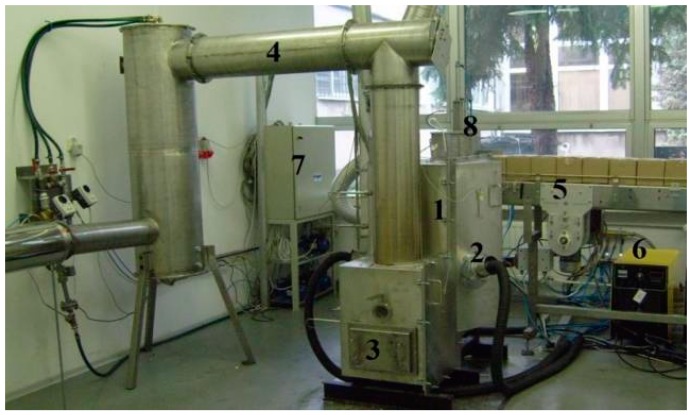
Overview of the laboratory setup: (1) plasma reactor; (2) plasmatron; (3) molten product collection; (4) fumes exhaust–chimney; (5) waste package transporter; (6) plasmatron power supply; (7) PLC-automation and data collection apparatus cabinet; (8) automatic waste package feeder.

In 2010, a research project was undertaken to investigate and design plasma technology that would allow processing of waste of printed circuit boards and recovery of the metals they contain. In the Industrial Research Institute for Automation and Measurements, the test setup was designed and tested. The stand is presented in Figure 2. The key component of the test setup is the plasma reactor, equipped with three plasmatrons-plasma sources, each located every 120° around the reactor chamber. The test setup is equipped with peripheral systems, that include measurement and control apparatus for data acquisition and control of the process during research.

**Figure 3:**
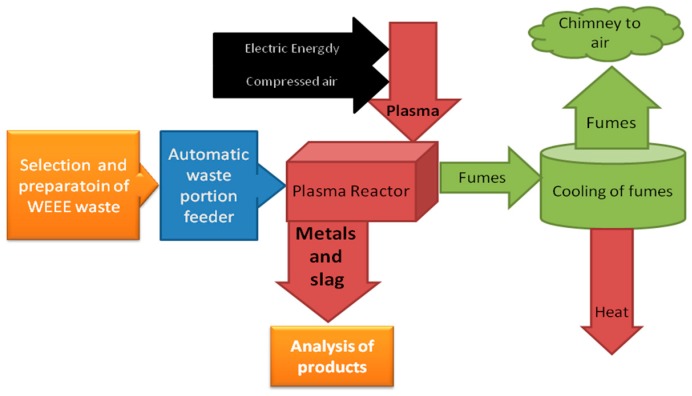
Block diagram of the designed process for investigations of high-temperature plasma technology for metals recovery and waste neutralization.

The designed test setup allows a wide range of experiments and data acquisition during investigations of waste processing and metals recovery. A block diagram of laboratory setup is presented in Figure 3.

**Figure 4:**
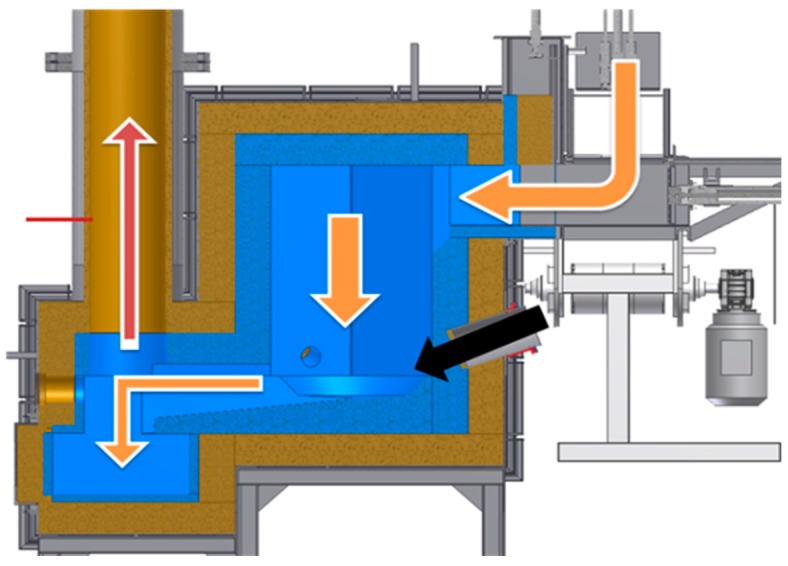
Cross-section through the plasma reactor with depiction of material flow. Waste and molten product–orange arrows, plasma stream–black arrow, fumes–red arrow.

The prepared waste portion is transported through an automatic feeder to the plasma reactor chamber. In the reactor chamber, the waste is incinerated and melted by three plasma streams. Next, the incineration fumes are transported to the scrubber where they are neutralized, cooled, and released to the atmosphere. As for the metals and slag, in the molten form they flow out from the reactor and set in casts, from which they can be recovered and recycled. Figure 4 presents a cross-section of the reactor. Arrows mark the waste and metals route (orange arrow), plasma stream (black arrow), and the direction of fumes exhaust (red arrow). The construction of the test reactor chamber allows immediate slag and molten metal outflow, so they are not retained in the reactor chamber. 

### 2.1. Reactor Construction

The reactor consists of three layers: first is fire-proof concrete, next is the thermal insulation, and last is the external metal construction shell. The reactor chamber is hexagonal, and its construction is presented in Figure 4. Such construction is capable of resisting temperatures of up to 1750 °C in its volume. However, in the area where the plasma streams have a direct effect on the waste, the temperatures exceed the temperature given above. 

### 2.2. Plasma Source 

The plasmatron plasma reactor has three sources of heat, which are 20 kW arc plasmatrons. The plasmatrons’ overall efficiency reaches 70%. Each plasmatron generates a stream of plasma that flows out from the bottom of the reactor chamber. The plasma is produced from compressed air, which is used as plasmatron working gas [16]. Three plasmatrons consume 11 Nm^3^/h of air during normal operation. 

### 2.3. Waste of Printed Circuit Boards Used in Experiments 

**Figure 5:**
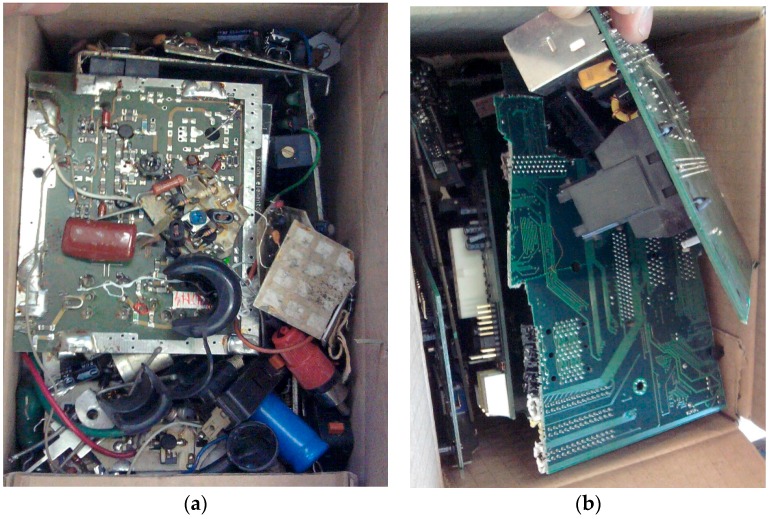
Waste printed circuit boards used in experiments. (**a**) Picture presents circuits and other mixed waste components used in experiment no. 2; (**b**) Picture presents PCB from personal computers used in experiments no. 1 and no. 3.

In the presented experiments, 36 kg of PCB waste was used in total (Table 3). The experiments were carried out in 2012 on printed circuit boards collected from used PCs and other electronic scrap (Figure 5). In the experiments, two types of PCB waste were used:

(1) In experiments no. 1 and no. 3, printed circuit boards from personal computers were used (Figure 5b). The waste was prepared by removing aluminium heat sinks and steel elements. No other components were removed.
(2) In experiment no. 2 the PCB waste used was of a different category (Figure 5a). Next to the PCBs from power supplies with transformers and other elements, PCBs from TV and radio equipment was used, and some old electrical and electronic components were added as well. Only large steel transformers and big aluminium heat sinks were removed.

The waste was prepared in the form of boxed portions, allowing its automated feeding into the reactor. Prepared waste portions, on the conveyor, are visible in Figure 2 (marked no. 5).

## 3. Results and Discussion

**Figure 6:**
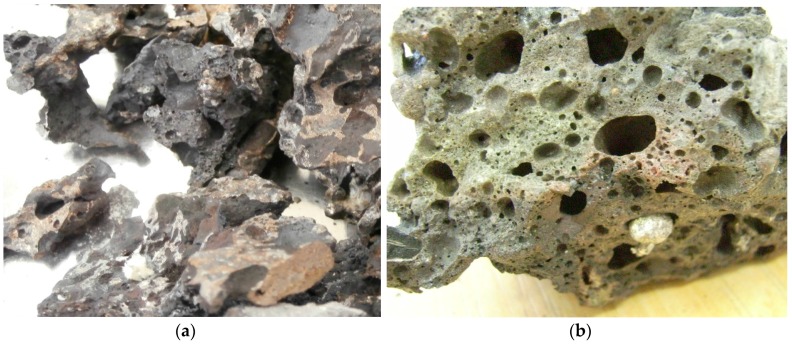
Photograph of separated products—metals (**a**); and slag (**b**).

Experiments were carried out by feeding portions of waste into the plasma reactor, which was preheated to a given temperature. During the experiments, the main process parameters were recorded continuously. After the process, the molten product was cooled, and its mass was measured. Then the metal fraction was separated from the slag (Figure 6), and their mass was measured. On the basis of collected data, process parameters were calculated. Summarized data of three experiments is presented in Table 4. During the experiments, some of the process parameters were varied to investigate their influence on the process performance. The time interval between each portion was changed, as was the amount of additional air for incineration, the temperature of the process, and the mass of the waste portion. 

Start and end temperatures indicate the temperature inside the reactor at the beginning of the experiment and at the end of the experiment (when the last waste portion was incinerated and melted), respectively. The difference between the start and end temperatures indicates the influence of incineration of additional fuel feed to the reactor in the form of PCB and cardboard. Data presented in Table 4 indicates that the temperature level highly affects the speed of the process. Experiments no. 1 and no. 2 were carried out at a starting temperature that was 50 °C higher than that for experiment no. 3. In both cases, the speed of the process was two times faster than in experiment no. 3. Compressed air was used for generation of plasma and incineration. Table 4 presents the amount of additional cold air being blown into the reactor in each experiment, and the plasmatrons’ air consumption. Energy consumption is calculated as overall electrical energy consumed by test setup, per kg of processed waste (kWh/kg). This value is calculated based on an average time of incineration and melting, for each individual waste portion, in each experiment. 

The important quality factors of waste processing technology are the mass reduction of waste and resource recovery level. The data presented in Table 4 specify that the waste mass reduction was achieved, changing from 60% to 80% of the input waste mass. Moreover 20%–27% of the input waste mass is the amount of the recovered metals. Therefore, the actual mass reduction is higher, and achieves a level of 40%–50% of the input waste mass. 

To calculate the metals recovery efficiency, recovered mass of metals from the three experiments presented in Table 3 was divided by the metals mass in PCB waste (31.5%—average from Table 1 and Table 2). The efficiency of metals recovery is 76%. Despite achieving a satisfactory level of waste mass reduction with this technology, the slag mass is still higher than expected. As it follows from the data presented in Table 1 and Table 2, the mass of the slag should be about 1/3 of the input mass, as the metals cover 31% of its mass, and the organic substance corresponds to another 30% of the waste mass. 

### 3.1. Increased Mass of the Slag 

Elemental analysis of the slag and metals (Table 5 and Table 6), produced during the plasma processing of printed circuit boards, reveals that some of the metals are being oxidized and are collected in the slag in the form of oxides. Calculating the iron and aluminium oxidation effect on slag mass increase demonstrates the effect of this mechanism. 

Assuming that only (1, 2) reactions take place:

2Fe + 3O → Fe_2_O_3_ − Energy (1)

Oxygen mass in Fe_2_O_3_ molecule—30.06%.

2Al + 3O → Al_2_O_3_ − Energy (2)

Oxygen mass in Al_2_O_3_ molecule—47.07%. 

Each 1% of oxidized Fe increases the slag mass by 1.3%, and each 1% of oxidized Al increases the slag mass by 1.47%. For example, the slag from experiment no. 3: Al_2_O_3_ mass in this slag is 17%, and the slag mass is 2.9 kg, which equals 0.493 kg of Al_2_O_3_ in the slag. Comparing to data from Table 1 and Table 2, where Al_2_O_3_ in PCB is 9.3% and aluminium 5.8%, calculating the input mass of the aluminium in PCB equals 0.039 kg, and the mass of Al_2_O_3_ equals 0.063 kg. In the process, aluminium becomes oxidized and its mass increases to 0.057 kg, so the total calculated mass of Al_2_O_3_ will be 0.12 kg. Comparing to the mass of the Al_2_O_3_ found in the slag, it is clear that PCB used in experiments had 3–5 times higher aluminium content then the samples presented in Table 1 and Table 2. Another factor is the aluminium content in the recovered metal. As it is shown in Table 5, the aluminium content in the recovered metal is very low (0%–0.36%). Calculation of the aluminium amount for experiment no. 3, using the average 0.22% and the metal mass 1.66 kg, a mass of 0.004 kg of Al is obtained. 

The above calculation of the oxygen bonded in metal oxides collected in the slag, explains 10%–20% of the slag mass increase. Furthermore, this mechanism is partially responsible for decreasing the mass of recovered metals. Expected metals recovery mass of about 30% decreases to 20%–27%. 

Worth noting is the SiO_2_ level in slag and in the PCB waste. In both cases, its level is very similar: 24.7% in PCB and 20%–27% in slag from the process. This means that regarding SiO_2_ content, PCB waste elemental composition varies only slightly. 

In the smelting industry, oxidation of metals is well known for Cu and Pb, where oxidation of impurities is the way to purify the main metal. However, in the presented plasma reactor there is no volume where the molten metal can be retained. The molten metal together with the slag flows out immediately after smelting, and sets in casts. That is why the metals have a very limited opportunity to mix and reduce, so they stay oxidized, i.e., 3.5%–7% of slag mass is Cu in the form of CuO and CuO_2_. To allow metals to mix together and reduce back from oxide to metal, a molten metal retention cavity is currently designed, and will be fitted in next experiments. 

### 3.2. Analysis of the Process Products: Metals and Slag

**Figure 7:**
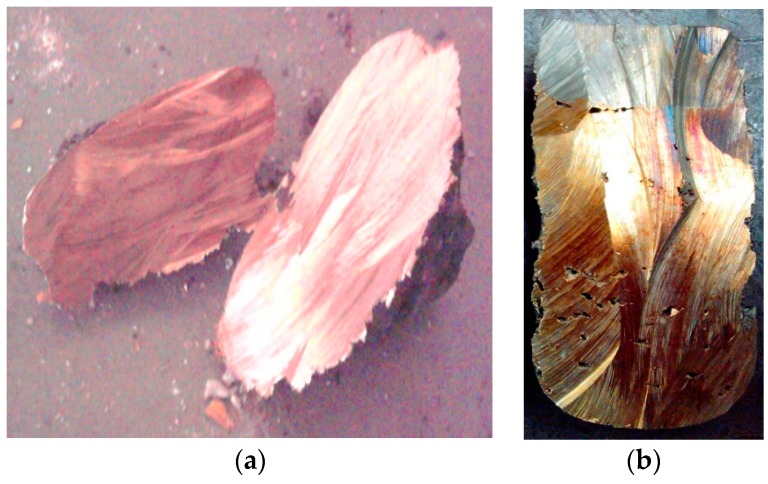
Photographs present surfaces of cut-through metal samples. Left picture (**a**) presents metal ingot from experiment no. 1. Right picture (**b**) presents remelted metal sample from experiment no. 3—visible segregation of metals into two layers, top (iron-rich) and bottom (copper-rich).

Solid product of plasma processing of PCB waste was segregated into two groups: metal and slag. Samples of both were sent for laboratory analysis to identify their chemical composition. Metals were remelted in small graphite crucible using electric arc, and left to set in. This homogenization procedure led to the formation of two layers in the ingot sample (Figure 7b). Top layer formed by iron-rich alloy (silver color), and bottom layer by copper-rich alloy (copper color). Samples were analyzed by microanalysis method—identification of elements in 0.01% of their occurrence. Analysis results are presented for both top and bottom layers for samples 2 and 3 (Table 5). Also in experiment no. 1, due to retention of molten metal in the reactor for 1 h after the experiment, the alloy was purified from Fe, Pb, Sn, and other metals and formed good quality ingot which consisted of over 91% Cu. (Figure 7a). Observed segregation of metals during the remelting procedure allows initial metals’ segregation. Top layer is formed by mostly Fe, 71%–78%, that collects most of Cr, Si, and some of Ni. Also 8%–17% of Cu is present in this layer, next to small amounts of other metals dissolved in this alloy. The bottom layer of ingot is formed mostly by Cu, 57%–76%, that collects Sn, Pb, Zn, Ca, Ag, and other metals in smaller amounts. Also, Fe is present in this layer at 6.7%–16%. 

In data from Table 5, an interesting observation is the low level of Pb in recovered metals. Pb was used with Sn in solder in older electric and electronic equipment—its usage was restricted by RoHS directive. On the other hand, the low Pb presence can be caused by its evaporation. The boiling point of Pb is 1740 °C [17]. This temperature level is easily achievable in a plasma reactor; additionally, the remelting procedure of the sample using electric arc (to homogenize the metals for elemental analysis), could decrease the amount of Pb in analyzed ingot samples. 

Slag samples were milled in a ball mill to homogenize the sample, and analyzed by the standardless XRF method. Results are presented in Table 6. It is assumed that every element detected in the slag is in the form of oxides. The XRF analysis does not identify in which form this element occurs: metal or oxide. Analyzing the results of elemental analysis of slag (Table 6), one can notice that the slag from experiments nos. 1 and 3 are quite similar. This similarity is due to the use of similar PCB waste in those experiments, i.e., PCB from personal computers. 

Analysis of the products of each experiment indicates that the slag from experiment no. 2 has lower SiO_2_ and CaO levesl, and higher Fe_2_O_3_, Pb, and Zn levels. This difference is caused by processing in experiment no. 2, together with PCB waste (different type than a PC), and loose electric and electronic components. This affects the waste composition, lowering the SiO_2_ and CaO amounts (mass of printed circuit boards), and increasing the amount of metals. Solder also contained more Pb than was found in samples from experiment No. 2. 

Interesting is the fact, identified by slag analysis, that the tantalum amount in it is 0%–0.11%. Also, Ag occurrence in slag (0.02%–0.07%) is interesting. Though, its presence can be explained by mixing of metals and slag in the current construction of the reactor chamber. 

## 4. Conclusions 

Processing of printed circuit boards with the presented plasma technology allows its effective neutralization and metals recovery. The composition of recovered metals and slag indicates that they are valuable raw resources. Overview of elemental analysis of recovered metals indicates that they can be used to recover copper, gold, silver, palladium, and others. Also, Sn recovery can be interesting as it occurs in reasonable amounts. As the metals segregate to Fe-rich fraction and Cu-rich fractions, Fe with Cr and Ni can be utilized. Further investigation is necessary to identify the content of other valuable metals, like the rare earth metals, in those products as some of them can be present in electronic printed circuit boards. 

Products’ composition will vary when different waste type is processed, however, the range of this variety can be calculated, and the products are repeatable and homogenous. 

Oxidation of metals can be exploited in order to pre-segregate the recovered metals, so the next refining steps can be shortened. Additionally, the evaporation of metals can be technologically interesting for removal and segregation—at high temperatures—of some of the metals occurring in PCB. 

Presented technology offers processing of PCB waste “as is”, without shredding it, and without any other preparation processes. This approach decreases the amount of energy required for waste processing and the number of process steps. It is estimated that energy consumption will be decreased even further due to optimization of reactor construction and other installation subsystems, which will also lead to an increase of the throughput of this technology. 

## Figures and Tables

**Table 1:** Estimated amounts of metal elements within printed circuit boards (PCB) [14].

Element	Share of PCB (Mass %)	Element	Share of PCB (Mass %)
Aluminium	5.8	Gold	0.023
Copper	9.7	Beryllium	0.003
Iron	9.2	Cadmium	0.014
Nickel	0.69	Chromium	0.052
Lead	2.24	Palladium	0.01
Tin	2.15	Bromine	2.03
Zinc	1.16	Chlorine	0.24
Silver	0.06	Antimony	0.35
Mercury	0.0009	**Total**	**33.8**

**Table 2:** Composition of the printed circuit boards from discarded personal computers [15].

Material	Element	Concentration (Mass %)	Total (Mass %)
Organic Epoxy Resin	C	18.10	31.8
	H	1.80	
	N	0.32	
	O (Org)	6.03	
	Br	5.07	
	Sb	0.45	
Inorganic Glass Fiber	SiO_2_	24.70	37.6
	Al_2_O_3_	9.35	
	CaO	3.36	
	MgO	0.081	
	BaO	0.0022	
	NaO	0.09	
	SrO	0.035	
Metal Circuit Solder	Cu	14.60	30.1
	Sn	5.62	
	Pb	2.96	
Lead Frame	Fe	4.79	
	Ni	1.65	
	Cr	0.356	
	Mo	0.016	
Contacts	Ag	0.045	
	Au	0.0205	
	Pd	0.022	

**Table 3:** Input waste mass in experiments.

Experiment No.	Mass of Waste Input (kg)		
	PCB	Cardboard	Average Portion
**1**	17.37	1.17	1.03
**2**	12.12	0.74	1.15
**3**	6.73	1.41	0.42

**Table 4:** Summarized results of processing of printed circuit boards in plasmatron plasma reactor.

Experiment No.	Products Mass (kg)		Products % Mass			Temperature (°C)		Air Consumption (Nm^3^/h)		Throughput (kg/day)	Energy Consumption (kWh/kg)
	Metals	Slag	Metals Recovery	Slag	Waste Reduction	Start	End	Plasma	Incineration		
1	3.53	7.12	20	41	61	1442	1492	11	15	586	2.66
2	3.27	6.58	27	54	81	1425	1560	11	11	724	2.06
3	1.66	2.9	25	43	68	1374	1553	11	6	312	4.99

**Table 5:** Elemental analysis of metal samples: composition of top and bottom layers in remelted ingots No 2, and No 3.

Element	No 1	No 2			No 3		
	% Mass	Sum % Mass	Top Layer %	Bottom Layer % Mass	Sum % Mass	Top Layer % Mass	Bottom Layer % Mass
**Cu**	**90.2**	**63.02**	8.41	76.41	**49.36**	17.00	57.29
**Zn**	**0.92**	**0.68**	0.00	0.85	**0.54**	0.18	0.63
**Pb**	**1.15**	**0.95**	0.51	1.06	**2.90**	1.00	3.36
**Sn**	**5.35**	**8.71**	0.78	10.66	**16.48**	5.21	19.24
**Fe**	**1.12**	**20.93**	78.67	6.77	**27.61**	73.26	16.42
**Ni**	**0.77**	**1.62**	2.34	1.44	**1.47**	1.75	1.40
**Al**	**0.01**	**0.12**	0.36	0.06	**0.07**	0.34	0.00
**Si**	**0.03**	**0.97**	2.05	0.70	**0.35**	0.86	0.22
**Ag**	**0.06**	**0.14**	n/a	**0.18**	**0.67**	**0.18**	**0.83**
**Au**	**0.0135**	**0.02**	n/a	**0.029**	n/a	n/a	n/a
**Pd**	**0.0048**	**0.01**	n/a	**0.015**	n/a	n/a	n/a

**Table 6:** X-ray fluorescence (XRF) analysis of slag samples from printed circuit boards processed in plasmatron plasma reactor.

Experiment No.	1	2	3
SiO_2_	27.81	20.02	21.85
TiO_2_	0.62	0.64	0.48
Al_2_O_3_	13.47	18.87	17.02
Fe_2_O_3_	13.65	21.01	12.68
MnO	0.41	1.81	0.35
MgO	0.34	0.3	0.24
CaO	12.6	6.05	8.13
K_2_O	0.09	0.14	0.07
P_2_O_5_	0.04	0.04	0.05
SO_3_	0.03	0.04	0.03
Ba	0.67	0.63	0.58
Co	0.01	–	0.01
Cr	0.13	0.12	0.68
Cu	3.5	3.77	7.03
Ni	0.19	0.19	0.35
Pb	0.32	1.23	0.35
Sr	0.1	0.07	0.05
Ta	–	0.09	0.12
Zn	0.47	1.16	0.74
Zr	0.07	0.07	0.05
Ag	0.02	0.07	0.02
Sb	0.11	0.04	0.17
Sn	1.07	1.39	1.47

## Abbreviations

The following abbreviations are used in this manuscript:

PCB	Printed Circuit Boards
WEEE	waste of electrical and electronic equipment
Mg	SI unit Mega Gram = tonne
